# BTK抑制剂联合苯达莫司汀、利妥昔单抗一线治疗慢性淋巴细胞白血病/小淋巴细胞淋巴瘤的疗效及安全性

**DOI:** 10.3760/cma.j.issn.0253-2727.2023.02.014

**Published:** 2023-02

**Authors:** 姝超 秦, 睿 姜, 业钦 沙, 婧妍 邱, 红岭 秘, 祎 缪, 微 吴, 莉 王, 磊 范, 卫 徐, 建勇 李, 华渊 朱

**Affiliations:** 1 南京医科大学第一附属医院，江苏省人民医院血液科，南京 210029 Department of Hematology, the First Affiliated Hospital of Nanjing Medical University, Jiangsu Province Hospital, Nanjing 210029, China; 2 江苏省人民医院浦口分院血液科，浦口慢淋中心，南京 211800 Pukou CLL Center, Pukou Division of Jiangsu Province Hospital, Nanjing 211800, China

慢性淋巴细胞白血病（CLL）/小淋巴细胞淋巴瘤（SLL）在西方发病率高，是成人最常见的白血病，我国的发病率低于西方，但呈不断增长趋势。小分子靶向药物布鲁顿酪氨酸激酶（BTK）抑制剂（BTKi）、促凋亡蛋白Bcl-2抑制剂（BCL-2i）维奈克拉（Ven）等治疗CLL/SLL具有疗效好、不良反应小、口服方便等特点[1]–[4]，显著优于传统化学免疫治疗，CLL治疗逐渐进入以BTKi为代表的无化疗时代[3]。我国已批准第一代BTKi伊布替尼及国产第二代BTKi泽布替尼、奥布替尼治疗CLL。BTKi单药治疗CLL缓解率高、生存期长，但由于缓解深度不高，很少达到完全缓解（CR），微小残留病（MRD）阴性者更少，所以BTKi单药需要长期治疗，长期治疗存在以下缺陷：经济负担、耐药相关的克隆演变、依从性差等。目前报道BTKi的主要耐药机制为BTK和磷脂酶C-γ2（PLC-γ2）基因突变等[5]–[6]。由于BTKi单药治疗的局限性，在既往疗效评估标准上结合以MRD阴性为治疗目标的新药联合或新药与传统化学免疫治疗（CIT）联合的固定周期治疗正逐渐成为研究的热点[6]–[9]。本研究分析报道本中心BTKi联合苯达莫司汀、利妥昔单抗（BR）方案一线治疗CLL/SLL的疗效及安全性。

## 病例与方法

1. 病例：纳入2020年1月至2021年11月期间在南京医科大学第一附属医院浦口慢淋中心就诊且满足治疗指征的9例初诊CLL/SLL患者（伦理编号：2021-NT-017），一线接受iBR（伊布替尼+BR方案）或zBR（泽布替尼+BR方案）治疗，其中iBR方案6例，zBR方案3例；CLL患者8例，SLL患者1例。CLL的诊断标准参照中国慢性淋巴细胞白血病/小淋巴细胞淋巴瘤的诊断与治疗指南（2022年版）[10]。CLL患者行外周血流式细胞术免疫分型、SLL淋巴结活检免疫组化检查。CLL患者的临床分期采用Binet和Rai分期。SLL患者的临床分期采用Ann Arbor分期标准。CLL患者采用国际CLL预后指数（CLL-IPI）评分系统进行预后评估[11]。

2. 治疗方案：伊布替尼420 mg每日1次或泽布替尼160 mg每日2次；利妥昔单抗第1个周期375 mg/m^2^，第2～6个周期500 mg/m^2^第0天；苯达莫司汀70 mg/m^2^第1～2天，所有患者第一阶段接受6个疗程iBR或zBR方案治疗，3个疗程及6个疗程后行全身增强CT或PET/CT、骨髓及骨髓活检进行疗效评估，同时行流式细胞术检测MRD，按计划完成iBR或zBR方案后采用伊布替尼/泽布替尼维持治疗。单药治疗阶段达2年时外周血和骨髓MRD维持阴性者按计划停药随访。联合方案治疗期间由临床医师判定不良反应与药物的相关性，并进行剂量调整，BTKi剂量调整主要依据用药说明，苯达莫司汀剂量调整主要依据药品说明及HELIOS试验研究者手册。

3. 疗效及安全性评价：CLL的疗效评估依据中国慢性淋巴细胞白血病/小淋巴细胞淋巴瘤的诊断与治疗指南（2022年版）[10]，SLL的疗效评估依据2014版Lugano标准[12]，评估总缓解率（ORR）、CR率、不良反应事件（AE）及MRD。AE按照CTCAE 5.0标准分级。MRD阴性定义为多色流式细胞术检测残存白血病细胞<1×10^−4^[13]。低MRD状态定义为残存白血病细胞≥1×10^−4^且<1×10^−2^，高MRD状态定义为残存白血病细胞≥1×10^−2^。

4. 随访：通过查阅患者住院病历确认患者治疗情况，对患者进行电话随访。末次随访日期为2022年1月31日，中位随访17.3（15.3～24.5）个月。

5. 统计学处理：计数资料采用例数描述，计量资料采用中位数（范围）描述。

## 结果

1. 临床特征：9例患者中位发病年龄54（43～72）岁，其中男8例，女1例。中位β_2_-微球蛋白3.6（2.6～4.5）mg/L，中位乳酸脱氢酶177（114～293）U/L，中位WBC 44.79（8.75～369.50）×10^9^/L，中位淋巴细胞计数绝对值37.7（0.6～131.5）×10^9^/L，中位HGB 121.5（64～153）g/L，中位PLT 88（43～225）×10^9^/L。CLL患者Binet分期：A期1例，B期2例，C期5例；Rai分期：Ⅰ期1例，Ⅱ期2例（25.0％），Ⅳ期5例（67.5％）。1例SLL患者Ann Arbor分期为Ⅳ期A组。IGHV无突变患者3例，del（17p）1例，del（11q）1例，del（13q）3例，TP53突变1例，NOTCH1突变1例，同时合并del（17p）和TP53突变1例。CLL-IPI评分低危（0～1分）、中危（2～3分）、高危（4～6分）、极高危（7～10分）患者分别为1、5、1、1例（表1）。

**表1:** 9例慢性淋巴细胞白血病（CLL）/小淋巴细胞淋巴瘤（SLL）患者的基线临床特征和疗效

例号	诊断	Binet分期	CLL-IPI	IGHV突变	TP53突变	治疗方案	中期评估	结束化疗后评估
1	CLL	C	3	有	无	iBR	PR	PR
2	CLL	C	3	有	无	iBR	PR	CR
3	CLL	C	3	有	无	iBR	PR	CR
4	CLL	B	8	无	有	iBR	PR	CRi
5	CLL	B	1	有	无	iBR	PR	CR
6	SLL	N/A	N/A	无	无	iBR	CR	CR
7	CLL	A	5	无	无	zBR	PR	CR
8	CLL	C	2	有	无	zBR	PR	CRi
9	CLL	C	3	有	无	zBR	CR	CR

**注** CLL-IPI：慢性淋巴细胞白血病国际预后指数；iBR：伊布替尼+苯达莫司汀+利妥昔单抗；zBR：泽布替尼+苯达莫司汀+利妥昔单抗；PR：部分缓解；CR：完全缓解；CRi：骨髓未恢复的完全缓解；N/A：不适用

2. 疗效：9例患者均完成6个周期的iBR或zBR方案进入维持治疗阶段。3个周期后中期评估ORR为100％（表1），在7例接受中期骨髓评估的患者中，CR 2例，部分缓解（PR）5例。4例患者外周血MRD阴性，其中2例外周血和骨髓均MRD阴性。2例未行骨髓评估的患者外周血均达到MRD阴性。6个周期后ORR为100％，CR 6例，骨髓未恢复的CR（CRi）2例，PR 1例。5例患者外周血达到MRD阴性，其中4例外周血和骨髓均达到MRD阴性。在单药维持阶段，截至末次随访，最佳外周血MRD阴性率为66.7％，外周血及骨髓MRD阴性率为55.6％，其中1例患者伊布替尼单药维持8个月后达到MRD阴性（图1）。例7在6个疗程后MRD>0.01％，在泽布替尼单药维持治疗4个月后外周血MRD 1.83％，自行选择联合维奈克拉治疗。例1在6个疗程后MRD>0.01％，在单药维持治疗2个月后因出现第二肿瘤（精原细胞瘤）而停用BTKi。例2因出现4级发热性中性粒细胞减少而停药并拒绝恢复单药治疗（停药时已达外周血MRD阴性）。其余患者仍口服BTKi单药，每3～6个月随访外周血MRD，均维持原有水平，最长随访时间24.5个月。

**图1:**
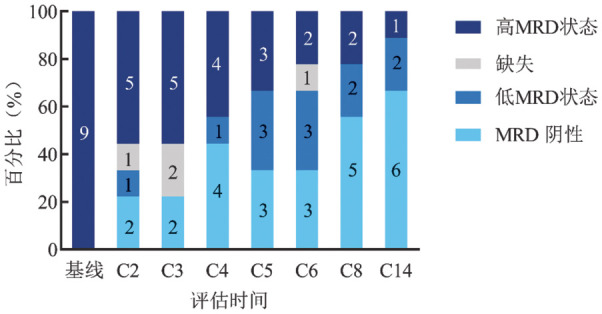
9例慢性淋巴细胞白血病/小淋巴细胞淋巴瘤患者治疗过程中外周血微小残留病（MRD）情况 **注** 图中数字表示例数；Cn表示第*n*个周期第1天

3. AE：最常见的血液学AE是血小板减少（9例）、贫血（7例）和中性粒细胞减少（6例）。最常见的非血液学AE是恶心（3例）、皮疹（3例）和乏力（2例）。其中3～4级AE包括贫血1例、中性粒细胞减少3例、发热性中性粒细胞减少2例、血小板减少2例，队列中未观察到3～4级非血液学AE。考虑和BTKi相关的AE包括血尿2例、齿龈出血1例及心房颤动1例。单药维持治疗期间，例1和例2分别由于伴发第二肿瘤、发热性中性粒细胞减少（4级AE）停止口服BTKi治疗。例3因伊布替尼单药维持治疗阶段出现反复症状性心房颤动，减量至140 mg每日1次仍无明显改善而更换为泽布替尼治疗，心房颤动未再出现。例4因反复发生齿龈出血而将伊布替尼减量至280 mg每日1次维持治疗。

## 讨论

CLL异质性很强，CLL-IPI已在预估一线CIT患者的总生存（OS）期方面得到验证，其中低风险患者的中位OS期未达到，而极高风险患者仅为29个月[11]。近二十年来，CLL的治疗发生了很大的变化。氟达拉滨、环磷酰胺和利妥昔单抗（FCR）方案成为65岁以下体能状态良好患者的标准一线治疗[14]–[15]。而CLL10的数据显示，BR方案在65岁以上的初治患者中不良反应较小且疗效尚可[16]。然而，具有不良生物学特征的患者，如IGHV未突变患者和TP53异常患者，未能从CIT中受益。以BTKi、BCL-2抑制剂和磷酸肌醇3-激酶抑制剂（PI3Ki）为代表的小分子靶向药物在初治和复发/难治性CLL中的应用部分克服了不利的生物学因素，使CLL的治疗模式进入了无化疗时代[17]–[20]。RESONATE-2研究提示，CLL患者应用伊布替尼单药随访7年后ORR为92％，疗效显著，但CR率仅为34％，MRD阴性率不到10％，缓解深度不够[21]。根据CLL10临床试验，尽管BR组中位PFS时间较FCR组缩短（43个月对54个月），但其3～4级中性粒细胞减少和感染的发生率也显著降低（分别为68％对88％、25％对40％），患者耐受性更好[16]。参考HELIOS研究[22]，本中心通过队列研究探索BTKi联合BR方案一线治疗CLL/SLL的疗效及安全性。

Dana-Farber癌症中心探索了伊布替尼联合氟达拉滨、环磷酰胺和利妥昔单抗（iFCR）方案治疗初治年轻CLL患者的有限周期的治疗模式，入组患者中46例（58.2％）无IGHV突变。截至中位随访时间40.3（3.1～76.0）个月时，CR或CRi率、骨髓MRD阴性率和外周血MRD阴性率分别为77.2％（44/57）、80.9％（50/62）和80.6％（55/68）。本研究入组患者中3例无IGHV突变，中位随访17.3（15.3～24.5）个月时CR或CRi率、骨髓MRD阴性率和外周血MRD阴性率分别为88.9％、55.6％和44.4％。其中未达CR或CRi的患者由于合并第二肿瘤在单药维持阶段退出评估。与iFCR方案相比，我中心iBR/zBR方案在随访时间较短的情况下CR/CRi率更高，骨髓及外周血MRD阴性率较低，随着单药维持和随访时间的延长，结果有待进一步观察。

MRD被认为是CLL患者10年PFS率的独立预测因素，与CIT时代的前线治疗无关[23]–[24]。MRD驱动的联合方案有望在CLL中实现有限疗程治疗，但停药时机仍有待探索。我们的队列研究发现，与中期评估相比，6个疗程后患者的ORR依然维持在100％，CR率及MRD阴性率均有所提升（分别为22.2％对66.7％、44.4％对55.6％），提示应用6个周期BTKi联合BR方案可提高ORR及CR率，且随着周期数增加，缓解程度可进一步加深。我们观察到1例应用6个周期iBR方案后外周血及骨髓MRD阳性的患者，在单药维持治疗8个月后达到外周血MRD阴性，提示部分患者有望通过单药维持治疗加深缓解深度。Dana-Farber癌症中心的iFCR队列研究中，伊布替尼单药维持治疗2年时骨髓MRD阴性者考虑停药。在骨髓MRD阴性状态下完成iFCR方案并开始伊布替尼维持治疗的61例患者中，有13例（21.3％）出现了骨髓MRD复阳，其中7例患者接受了伊布替尼单药治疗，并在单药治疗后均达到PR。此外，MD Anderson癌症中心也进行了iFCG（伊布替尼+氟达拉滨+环磷酰胺+奥妥珠单抗）方案治疗初治CLL患者的有限疗程研究，计划治疗12个周期后骨髓MRD阴性的患者停止所有治疗，骨髓MRD阳性患者继续伊布替尼治疗[25]。中位随访30.2个月，第12个周期治疗后，73％的患者取得了CR或CRi，100％患者获得了骨髓MRD阴性，41例患者停药，中位随访18.7个月，无MRD复阳、疾病进展或Richter转化。关于有限疗程治疗方案中何时停药的探索仍在进行，需要进一步扩大样本量及延长随访时间以获得更充足的依据。

AE方面，我们的队列研究发现，随着治疗疗程数增加，3～4级AE中，只有1例患者持续出现中性粒细胞减少，而贫血、血小板减少及发热性中性粒细胞减少在后4个周期并未增多。提示随着疗程数的增加，严重AE的发生率并未增加，该方案安全性良好。在我们的队列中，所有患者均能耐受6个周期的化疗并进入单药维持治疗阶段，提示化疗期间不良反应较少，患者耐受性良好。与HELIOS研究相比，本队列的患者在前6个周期的化疗中3～4级AE发生率较低，没有患者在化疗阶段因为BTKi不良反应停药。考虑HELIOS研究纳入的患者为既往至少接受过一线治疗的复发/难治患者，我们考虑BTKi联合BR方案在初诊CLL患者中耐受性更好，安全性可控。

本研究仍存在入组病例数较少，随访时间较短等局限性，未来将延长随访时间，在MRD的指导下探索停药时机，实现有限疗程治疗。综上所述，本研究提示，在初诊CLL患者中，BTKi联合BR方案缓解深度良好，不良反应较少，耐受性可控，可作为初诊CLL患者有限疗程探索的治疗选择之一。
